# Diversity and Evolution of Novel Invertebrate DNA Viruses Revealed by Meta-Transcriptomics

**DOI:** 10.3390/v11121092

**Published:** 2019-11-25

**Authors:** Ashleigh F. Porter, Mang Shi, John-Sebastian Eden, Yong-Zhen Zhang, Edward C. Holmes

**Affiliations:** 1 Marie Bashir Institute for Infectious Diseases and Biosecurity, Charles Perkins Centre, School of Life & Environmental Sciences and Sydney Medical School, The University of Sydney, Sydney, NSW 2006, Australiamang.shi@sydney.edu.au (M.S.); js.eden@sydney.edu.au (J.-S.E.); 2 Centre for Virus Research, Westmead Institute for Medical Research, Westmead, NSW 2145, Australia; 3 Shanghai Public Health Clinical Center and School of Public Health, Fudan University, Shanghai 201500, China; zhangyongzhen@shphc.org.cn; 4 Department of Zoonosis, National Institute for Communicable Disease Control and Prevention, Chinese Center for Disease Control and Prevention, Changping, Beijing 102206, China

**Keywords:** DNA virus, invertebrates, meta-transcriptomics, evolution, circovirus, herpesvirus, contamination

## Abstract

DNA viruses comprise a wide array of genome structures and infect diverse host species. To date, most studies of DNA viruses have focused on those with the strongest disease associations. Accordingly, there has been a marked lack of sampling of DNA viruses from invertebrates. Bulk RNA sequencing has resulted in the discovery of a myriad of novel RNA viruses, and herein we used this methodology to identify actively transcribing DNA viruses in meta-transcriptomic libraries of diverse invertebrate species. Our analysis revealed high levels of phylogenetic diversity in DNA viruses, including 13 species from the *Parvoviridae, Circoviridae*, and *Genomoviridae* families of single-stranded DNA virus families, and six double-stranded DNA virus species from the *Nudiviridae*, *Polyomaviridae*, and *Herpesviridae*, for which few invertebrate viruses have been identified to date. By incorporating the sequence of a “blank” experimental control we also highlight the importance of reagent contamination in metagenomic studies. In sum, this work expands our knowledge of the diversity and evolution of DNA viruses and illustrates the utility of meta-transcriptomic data in identifying organisms with DNA genomes.

## 1. Introduction

Invertebrates harbour an enormous diversity of RNA viruses that are ancestral to many of those found in vertebrates [1,2]. Far less is known, however, about the diversity of DNA viruses carried by invertebrates. As with RNA viruses, known DNA viruses represent a tiny fraction of the total DNA virosphere, in part reflecting a strong sampling bias towards viruses that are clinically or economically relevant. Although there has recently been an effort to characterise more invertebrate RNA viruses [1], as well as studies documenting an abundance of DNA bacteriophage [3,4], there is relatively little information on the biodiversity of DNA viruses in these copious host organisms. 

The advent of metagenomic next-generation sequencing has transformed virus discovery [1,5], expanding our knowledge of the diversity of viruses in many environments [6], and providing rich information on virus evolution [1,5,7]. Importantly, the novel viruses detected through metagenomics have routinely been validated by confirmatory PCR [1]. Many DNA viruses have been identified through metagenomic methods, particularly shotgun sequencing of purified viral particles and rolling cycle amplification [6,8,9], although this is biased toward small circular genomes such as those found in single-strand (ss) DNA viruses [10,11]. Of particular note has been the metagenomic discovery of many novel and highly diverse species of circular single stranded (CRESS) DNA viruses [9,12,13,14,15]. Although total shotgun RNA sequencing, or meta-transcriptomics [5,16], has less often been used for DNA virus discovery [17], it is viable as long as the viruses in question are expressing RNA. While the reliance on active transcription means that some DNA viruses will necessarily be missed, meta-transcriptomics provides an in-built indication of the abundance of viral reads in the data and has the ability to explore the whole virome within a specific host, identifying those that are highly divergent or at low abundance. A major complicating factor, however, is that ssDNA viruses, particularly circoviruses and their relatives, are common contaminants of reagents, leading to false virus discovery and inferences on host associations [18,19]. 

Despite their obvious abundance, relatively little is known about the phylogenetic diversity of DNA viruses, particularly in the estimated more than 10 million invertebrate species on earth [20]. Herein, we aim to better understand the diversity and evolution within the DNA virosphere by documenting viruses present in meta-transcriptomic data from ~220 species across nine invertebrate phyla [1], as well as in newly acquired metagenomic data.

## 2. Materials and Methods 

### 2.1. Sample Collection, RNA Extraction, Library Preparation, and Sequencing

As described previously [1], invertebrate species were sampled from various terrestrial and freshwater locations (Anhui, Beijing, Hubei, Xinjiang, Zhejiang provinces) and marine and coastal (East and South China sea) environments in China. This resulted in the generation of 87 sequencing libraries of invertebrate samples covering a wide diversity of phyla, including Arthropoda (the majority), Nematoda, Platyhelminthes, Mollusca, Sipuncula, Annelida, Chordata, Echinodermata, and Cnidaria. In addition, a further five sequencing libraries were generated as part of this study, sourced from Arthropoda, Maxillopoda, Oliochaeta, Turritella and *Plasmodium berghei* (Supplementary Table S1). The processed samples were organized into either simple (single/multiple samples from one or two closely related species) or complex (multiple species from a taxonomic group) libraries that we then mined for DNA viruses. Total RNA was extracted from the samples, and ribosomal RNA (rRNA) was depleted during the library preparation step. Each library underwent paired-end (90 or 100 bp) sequencing on a HiSeq2000 platform (Illumina, San Diego, USA). A more detailed description of the collection, storage, processing, construction, and sequencing of the libraries can be found elsewhere [1].

In the analysis of the *Circoviridae*, an additional sequence was included from an experimental blank control derived from previous experiments performed by our group (https://www.ncbi.nlm.nih.gov/sra/SRX6803604). This blank control, representing sterile water with reagents, contained a circovirus-like sequence. This sequence, along with reagent contamination-associated circovirus sequences identified previously [18], was used to place the true diversity of circoviruses within the context of what might be expected from reagent contamination. An equivalent analysis was performed using available densovirus sequences that also represent likely reagent contaminants [18].

### 2.2. Sequence Assembly and Virus Discovery

Each library had its respective sequencing reads trimmed and assembled de novo using the Trinity program with default settings [21]. Trinity-assembled contigs were used in a comparative analysis using diamond BLASTX against the GenBank non-redundant (nr) database [22]. A threshold e-value of 1 × 10^−5^ was used to minimize false-positives while maintaining sensitivity. Taxonomic information was collated for candidate novel virus sequences using the in-house Taxonomist program (Buchmann et al., in preparation) that determines the predicted taxonomy for each candidate virus. Conserved domains were predicted using the NCBI conserved domain database (CDD) with a threshold *e*-value of 1 × 10^−1^. To determine how abundant viral reads were within each library we calculated their percentage within the total number of reads in a library (that necessarily excludes ribosomal RNA reads) by mapping the trimmed reads back to the assembled contigs. While the read levels generated by this method cannot be individually used to determine the validity of a putative virus, in combination with robust phylogenetic inference this approach is an effective means to discover novel virus sequences [1,2].

### 2.3. Phylogenetic Analysis 

To facilitate phylogenetic inference among six DNA virus families, we collected amino acid sequences from highly conserved reference proteins for each DNA virus family under consideration. Accordingly, amino acid sequences of each reference protein from the NCBI Refseq database were downloaded for each family: the viral replicase initiation protein (Rep) for the *Genomoviridae* (*n* = 39) and *Circoviridae* (*n* = 82), the non-structural protein (NS1) for the *Parvoviridae* (*n* = 69), the large T antigen protein for the *Polyomaviridae* (*n* = 84), the anti-apoptosis protein for the *Nimaviridae* (*n* = 5), the DNA polymerase for the *Nudiviridae* (*n* = 3), and the DNA packaging subunit 1 for the *Herpesviridae* (*n* = 37). These sequences were aligned with the respective novel viral sequences using the E-INS-i algorithm in MAFFT v7 [23,24]. Ambiguously aligned and unaligned regions were removed using GBlocks [25,26]. Final sequence alignments (i.e., following GBlocks pruning) were all between 100–300 amino acids in length. ProtTest v3.4 [27] was used to determine the best-fit model of amino acid substitution for each alignment (Supplementary Table S2). Phylogenetic trees of these alignments were then estimated using the maximum likelihood method available in PhyML version 3.0 [28], employing the Subtree Pruning and Regrafting (SPR) branch-swapping algorithm and a bootstrap resampling analysis (1000 replications). 

## 3. Results

### 3.1. Data Mining for DNA Viruses

**Figure 1:**
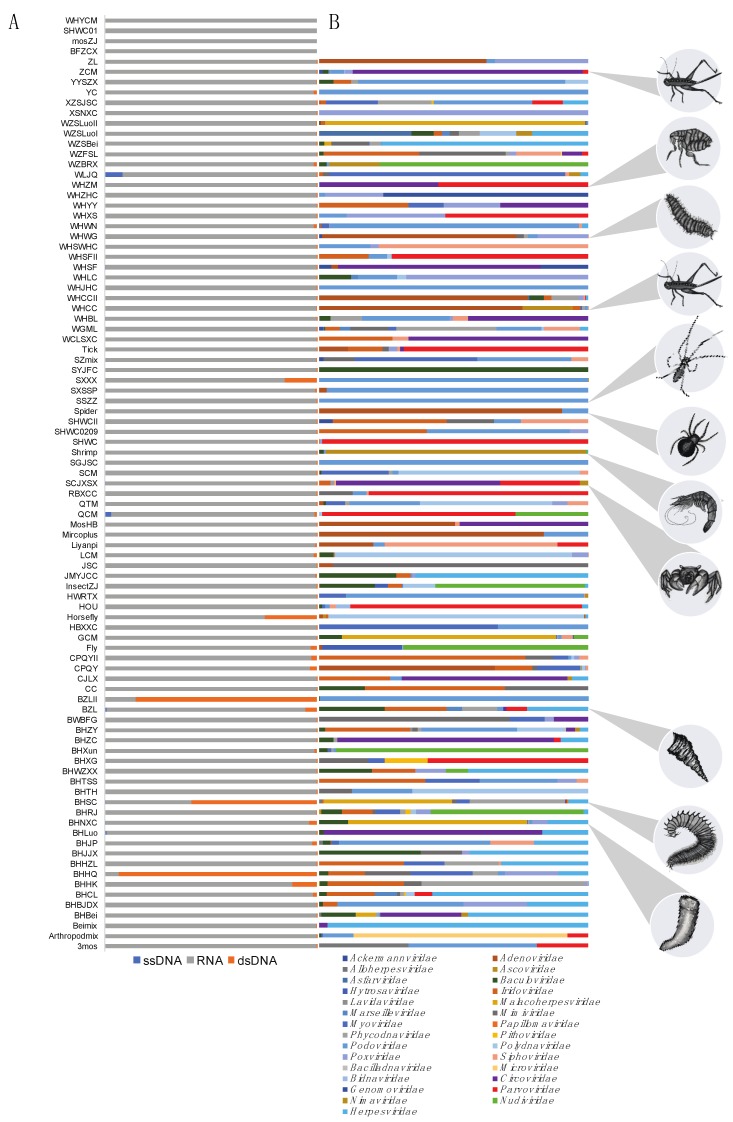
Composition and abundance of virus-like contigs in the 92 RNA sequencing libraries analysed here. (**A**) The relative abundance of RNA, ssDNA, and dsDNA virus-like contigs in all 92 libraries, calculated by mapping the contigs back to reads and shown as a percentage of total reads (excluding ribosomal RNA). (**B**) The abundance of expressed genes in DNA viruses (separated by family) within the libraries. The source (host) of the libraries where novel DNA viruses were identified are depicted with grey circles.

We screened meta-transcriptomic data from a wide range of invertebrate phyla and species for candidate DNA virus sequences. These meta-transcriptomic data had previously been shown [1] to be rich in RNA viruses (Figure 1A). Our analysis revealed the presence of 16 novel DNA virus sequences from a range of virus families as well as three new variants of known virus species (Table 1). Although the presence of these novel viruses will need to be confirmed by future PCR, that we identified variants of known viruses at relatively high abundance, such as Periplaneta fuliginosa densovirus variant Hubei (see below and Table 1), suggests at our meta-transcriptomic approach is an effective means to identify actively transcribing DNA viruses.

In total, 12 novel species and one variant were identified from the ssDNA families *Genomoviridae*, *Circoviridae*, and *Parvoviridae* (Figure 1B; Table 1). Although DNA virus-like hits (i.e. those identified as DNA viruses using the Taxonomist program) were identified from a diverse range of virus families, many were likely false-positives or very short (<400 bp) and so could not be confirmed with confidence. Novel and divergent gemykibivirus-like sequences are referred to here as “gemy-like viruses”, while densovirus-like sequences are termed “denso-like viruses”, and circovirus-like viruses as “circo-like viruses”. In addition, five novel species of dsDNA viruses and a potentially novel virus variant were identified from the *Polyomaviridae, Nudivridae, Nimaviridae*, and *Herpesviridae*. The sequences detected include a “polyoma-like” virus sequence, two “herpes-like” virus sequences, two “nudi-like” virus sequences, and a “nima-like" virus sequence that was very similar to white spot syndrome virus (WSSV) (Table 1). The abundance of each novel DNA virus, itself dependent on levels of gene expression, was estimated as the percentage of reads mapped to a viral genome, divided by the total number of reads in the library (from which rRNA has been excluded): this resulted in a measure of the percentage of DNA virus-like sequence reads in each library. We now describe each of the novel DNA virus sequences in turn.

### 3.2. Diversity and Phylogenetic Relationships of Novel ssDNA Viruses

#### 3.2.1. *Genomoviridae*

Three novel gemy-like sequences were identified in the WGML library sampled from multiple individuals of the Myriapoda phylum (millipedes; Table 2), sampled from Beihai, China. The first gemy-like sequence, that we term Myriapoda gemykibivirus 1, is 2250 bp in length and made up 0.004% of the (non-rRNA) reads in the library. Similarly, another gemy-like sequence, termed Myriapoda gemykibivirus 2 (2286 bp) comprised 0.0005% of reads, while Myriapoda gemykibivirus 3 (2324 bp) made up 0.005% of the reads. All sequences exhibit 46%–66% amino acid identity to gemycircularvirus-specific virus proteins, including the highly conserved replication protein (Rep) and the capsid protein (Table 1). In addition, when the three sequences were translated, each amino acid sequence mapped to the highly conserved AL1 domain present in *Genomoviridae*.

**Figure 2:**
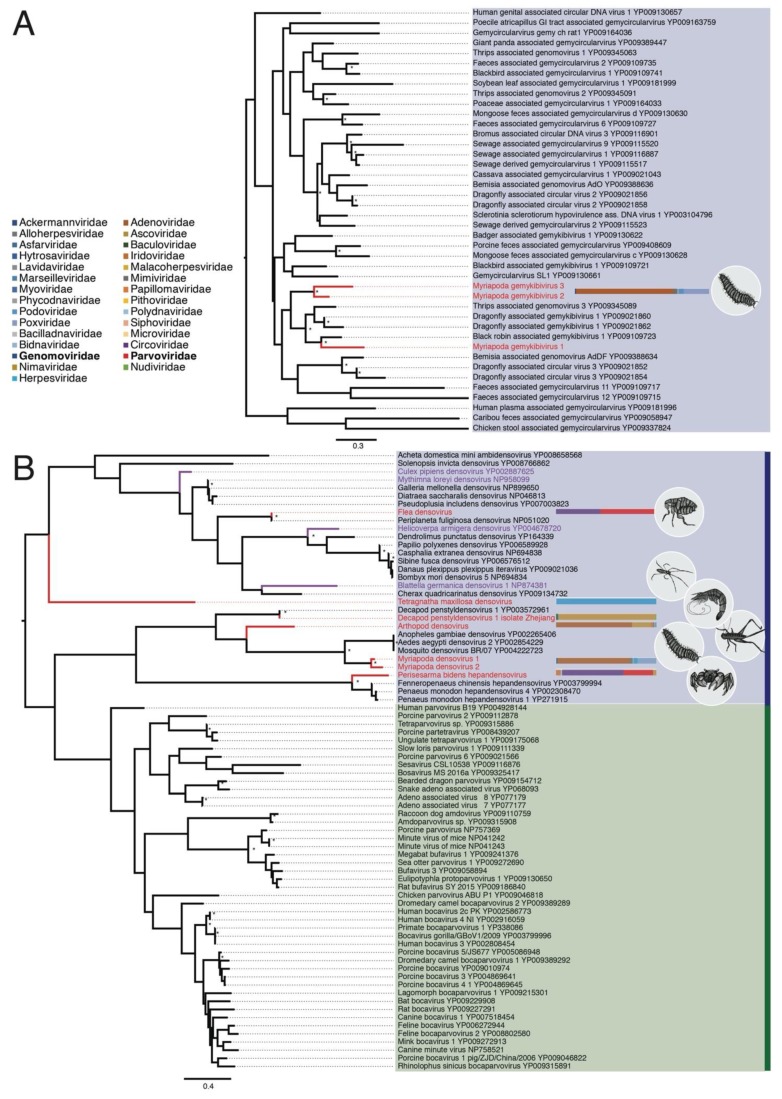
Phylogenetic relationships of the ssDNA virus families *Genomoviridae* and *Parvoviridae*, including the novel viruses identified in this study. The abundance of family-specifc contigs are represented by horizontal bars next to the associated virus. This representation of viral contig abundance is limited to the DNA virus families for clarity. (**A**) Phylogenetic relationships of gemykibiviruses, including three novel species discovered here: Myriapoda gemykibivirus 1, Myriapoda gemykibivirus 2 and Myriapoda gemykibivirus 3 (highlighted in red). All three viruses were sampled in millipedes (Arthropoda, myriapoda) from Beihai, China (WGML library). For clarity, the tree was mid-point rooted. The abundance of gemy-like contigs (blue portion) is shown beside the novel sequences. Bootstrap values greater than 85% are shown next to nodes, represented by an asterisk. All horizontal branch lengths are scaled according to the number of amino acid substitutions per site. (**B**) Phylogenetic relationships of the *Parvoviridae*, including the seven densoviruses identified in this study: Perisesarma bidens densovirus, Arthropod densovirus, Myriapoda densovirus 1, Mydriapoda densovirus 2, Decapod penstyldensovirus 1 variant Zhejiang, Tetragnatha maxillosa densovirus, and Periplaneta fuliginosa densovirus variant Hubei (highlighted in red). Several densovirus-like sequences that have been previously associated with reagent contaminantion were also included and highlighted in purple. The tree was mid-point rooted to distinguish the *Densovirinae* and the *Parvovirinae* (blue and green boxes, respectively). The abundance of parvo-like contigs (represented by the red portion) in the respective libraries is shown beside the two novel sequences. Bootstrap values greater than 85% are shown next to the relevant nodes, represented by an asterisk. All horizontal branch lengths are scaled according to the number of amino acid substitutions per site.

As all three novel gemy-like sequences contained a hypothetical Rep sequence, we aligned them to the amino acid sequences of 39 Rep sequences from the Genomoviridae available on Genbank. A phylogenetic tree of these data revealed that the three sequences represent novel gemykibiviruses that fall within a (weakly supported) clade of genomoviruses and gemykibiviruses identified in other invertebrates, including Thrips-associated genomoviruses and Dragonfly-associated gemykibivurses (Figure 2A). Black robin-associated gemykibivirus 1 also falls in this clade.

#### 3.2.2. *Parvoviridae*

Seven novel denso-like sequences were identified in six libraries. The first, termed Decapod penstyldensovirus 1 variant Zhejiang (3137 bp) as it is closely related to other Decapod pentstyldensovirus 1 sequences (Supplementary Figure S3a,b), has abundant reads within the Shrimp library (0.02% of library reads) (Table 2). Two novel denso-like sequences were sourced from the WGML library (from millipedes): Mydriapoda densovirus 1 (739 bp) and Mydriapoda densovirus 2 (2460 bp) (0.0006% and 0.001% of library reads, respectively). Another denso-like sequence, termed Periplaneta fuliginosa densovirus variant Hubei (1658 bp) because it is very closely related to Periplaneta fuliginosa densovirus, had highly abundant reads in the flea and ant mix WHZM libraries (0.27%), and had high identity to the conserved non-structural protein 1 (NS1) region (Table 1). Arthropod densovirus (970 bp), present in the WHCC library (0.0001% of reads) and sourced from a mix of insects, exhibited sequence similarity to the NS1 domain of other densoviruses. The final two denso-like sequences identified were Perisesarma bidens hepandensovirus (940 bp), detected in a library representing a sesarmid crab mix, SCJXSX (0.0002% of reads), and Tetragnatha maxillosa densovirus (1087 bp) present in the SSZZ library (0.002% of reads) from a *Tetragnatha maxillosa* mix.

All of the seven parvo-like sequences contained regions of high similarity to densovirus proteins, including the highly conserved non-structural protein 1 (NS1) and structural proteins (Table 1). These conserved NS1 sequences were then aligned with homologous sequences from the Parvoviridae available on GenBank (*n* = 69) and used in a phylogenetic analysis. All seven sequences fell within the sub-family Densovirinae known to infect invertebrates (Figure 2B). The newly described Perisesarma bidens hepandensovirus falls basal to Penaeus monodon hepandensoviruses 1 and 4, and Fenneropeneus chinensis hepandensovirus. Arthropod densovirus falls basal to the clade that includes two other novel denso-like sequences, Mydriapoda densovirus 2 and Mydriapoda densovirus 1, as well as mosquito-infecting densoviruses. Arthropod densovirus was found in the WHCC library, comprised of a mix of insect species (Table 2—*Pseudothemis xonata, Nepidae sp, Componotus japonicas, Diponychus sp., Asellus sp.*), making it difficult to determine the true host. The two denso-like sequences from the WGML library (from millipedes), fall in the same clade as mosquito-infecting densoviruses Anopheles gambiae densovirus, Mosquito densovirus BR/07, and Aedes aegypti densovirus 2. As reflected in its name, Decapod penstyldensovirus 1 variant Zhejiang is very closely related to another crustacean-infecting densovirus, Decapod penstyldensovirus 1. When the NS1 region of these two sequences were aligned against each other they shared 96% amino acid identify (Supplementary Figure S3a), and when used in a whole genome alignment with Decapod penstyldensovirus 1, Decapod penstyldensovirus 1 variant Zhejiang shared 96.14% of nucleotide identity (Supplementary Figure S3b). This suggests that this sequence is a novel variant of this species. Tetragnatha maxillosa densovirus is extremely divergent, falling basal to the second major clade of densoviruses that includes Periplaneta fuliginosa densovirus variant Hubei. As Periplaneta fuliginosa densovirus variant Hubei is very closely related to Periplaneta fuliginosa densovirus (associated with cockroaches), at both the nucleotide and amino acid levels (98%–100% identity), this sequence is likely a novel variant of this virus species. Finally, several densovirus sequences that have previously been associated with reagent contamination [18] were included in this analysis—Blattella germanica densovirus 1, Culex pipiens densovirus, Helicoverpa armigera densovirus, and Mythimna loreyi densovirus (highlighted in purple in Figure 2B). None of these contaminant sequences were closely related to the novel densoviruses identified here. 

#### 3.2.3. *Circoviridae*

Three novel circo-like genomes were identified from three different libraries in the meta-transcriptome data set (Table 1): (i) Sandworm circovirus (1063 bp), present in BHSC (0.0004% of reads) sourced from sandworms; (ii) Arthropod cyclovirus (1911 bp), with highly abundant reads in the ZCM library (0.02% of reads) derived from a mix of insects; and (iii) Flea cyclovirus (1602 bp) that had highly abundant reads (0.87% of reads) in the WHZM library, obtained from a majority Ctenocephalides felis flea and ant mix from Hubei, China. A fragment (1386 bp) of Flea cyclovirus was also identified at low abundance (0.00008%) in the Arthropodmix library, representing a mix of Arthropod species (Table 2). All three novel circo-like sequences contained regions that were highly similar to the conserved Circoviridae replication protein (Rep) (Table 1).

**Figure 3:**
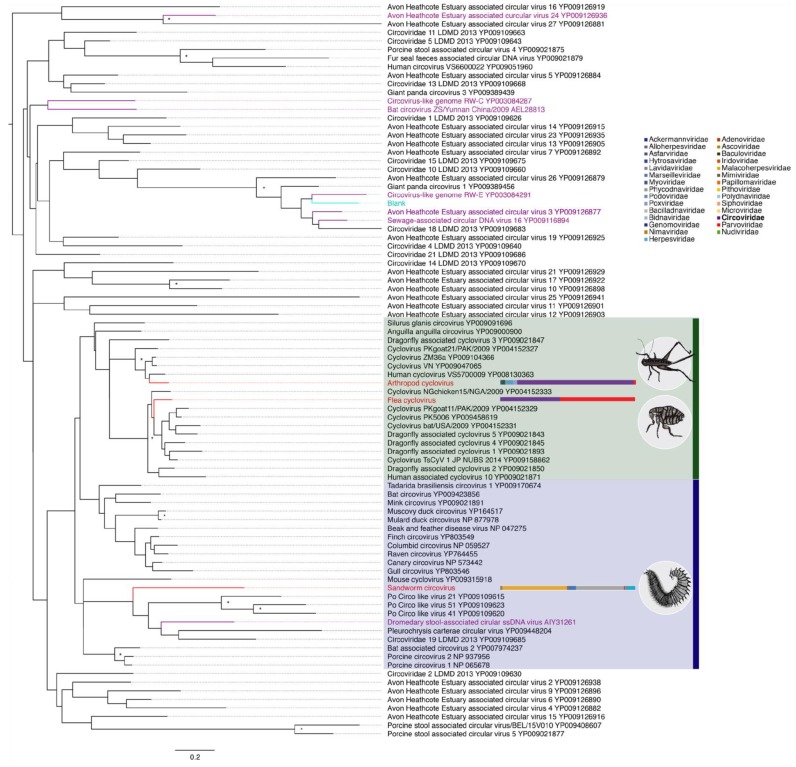
Phylogenetic relationships of *Circoviridae* sequences, including the three novel species identified in this study: Arthropod cyclovirus, Flea cyclovirus, and Sandworm circovirus, sourced from the ZCM, WHZM and BHSC libraries, respectively (highlighted in red). The novel Flea cyclovirus falls within the predominantly cyclovirus clade, shown in the green box, while Sandworm circovirus falls in a diverse circo-like virus clade that includes a likely reagent contaminant (blue box). A circo-like sequence obtained from an experimental sterile water control, denoted ’Blank’, is also included (highlighted in blue) and falls within a diverse range of faeces and marine-associated circoviruses, as do most circovirus sequences previously suggested to represent reagent contaminants (highlighted in purple). The tree was mid-point rooted for clarity only. The abundance of circo-specifc contigs are represented by the purple portion in the horizontal bars next to each circo-like virus. This representation of circo-like contig abundance is limited to DNA virus families for clarity. Bootstrap values greater than 85% are shown next to the appropriate nodes, represented by an asterisk. All horizontal branch lengths are scaled according to the number of amino acid substitutions per site.

The amino acid sequence of the three novel circo-like sequences mapped to the highly-conserved Rep protein. We performed a phylogenetic analysis of these sequences, other members of the *Circoviridae* collated from GenBank (*n* = 82), and a blank sequence that includes a circo-like fragment found in an experimental control sample used to identify reagent contaminants. Two novel circo-like sequences, Flea cyclovirus and Arthropod cylcovirus, fell within the cyclovirus branch and are related to other known invertebrate-infecting circoviruses, including several Dragonfly-associated cycloviruses, while Sandworm circovirus falls within a more diverse clade of circo-like viruses (Figure 3). The remaining clades of this phylogeny comprise a diverse set of viruses largely associated with faeces, ocean- or estuary-associated samples. Notably, the Blank circo-like sequence (highlighted in blue) and the previously identified [18] circovirus-like sequences associated with reagent contamination (highlighted in purple) also fall within these clades, with the single exception of a Dromedary stool associated circovirus. 

### 3.3. Discovery and Phylogenetic Relationships of Novel dsDNA Viruses

#### 3.3.1. *Herpesviridae*

**Figure 4:**
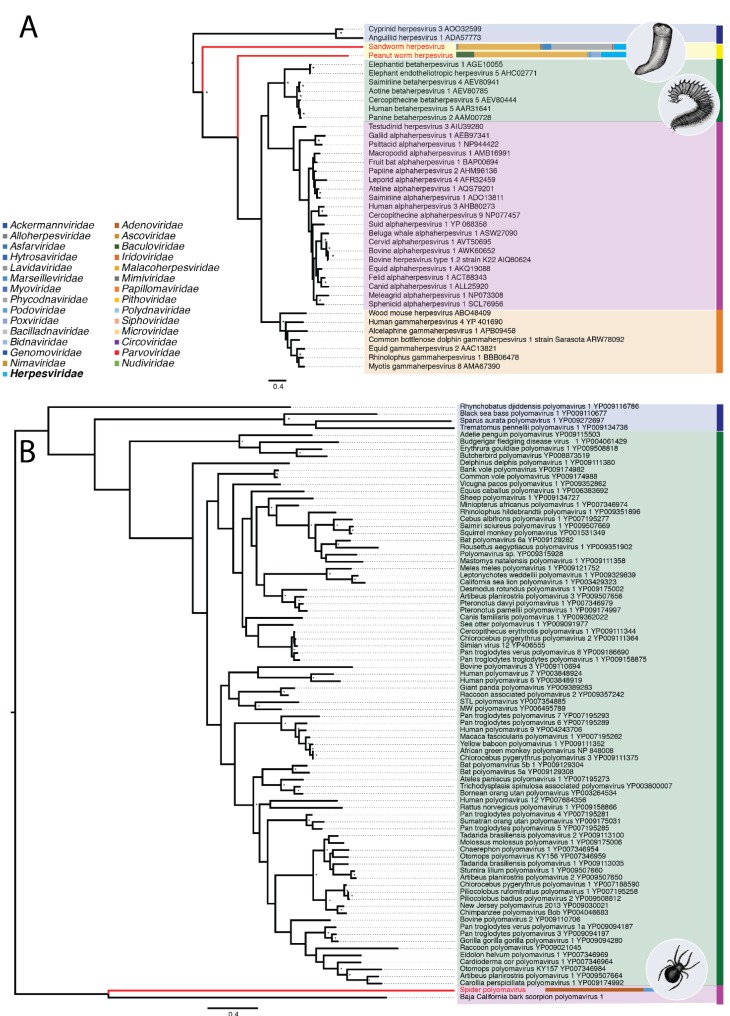
Phylogenetic relationships of the dsDNA virus families *Polyomaviridae* and *Herpesviridae*, including the novel species identified in this study. The abundance of family-specifc contigs are represented by horizontal bars next to the associated virus. This representation of viral contig abundance is limited to the DNA virus families for clarity. (**A**) Phylogenetic relationships among known herpesviruses and the two novel sequences, Peanut worm herpesvirus (BHNXC library) and Sandworm herpesvirus (BHSC library) identified here and highlighted in red. The mollusc-infecting Malacoherpesviruses are shown in the blue box. Alpha-, beta- and gamma-herpesviruses are shown in the green, pink, and orange boxes, respectively. The two novel herpesvirus sequences identified here are shown in yellow. The abundance of herpes-like contigs (represented by the light blue portion) is shown beside the two novel sequences. Bootstrap values greater than 85% are shown next to appropriate nodes, marked by asterisks. All horizontal branch lengths are scaled according to the number of amino acid substitutions per site. For clarity, the tree is mid-point rooted. (**B**) Phylogenetic relationships among 88 polyomaviruses including the novel Spider polyomavirus (highlighted in red). The novel Spider polyomavirus falls in a distinct clade (pink box) that includes the single other invertebrate-infecting polyomavirus, Baja California bark scorpion polyomavirus 1. This clade is distinct from the fish-infecting polyomaviruses (blue) and terrestrial polyomaviruses (green). Bootstrap values greater than 85% are shown next to their appropriate nodes, marked by an asterisk. As polyoma-like virus reads were at low abundance in all libraries their abundance is not shown here. All horizontal branch lengths are scaled according to the number of amino acid substitutions per site. For clarity, the tree was mid-point rooted.

The BHSC and BHNXC libraries (Table 2) both yielded herpes-like virus sequences. Specifically, the two novel viruses were identified in two worm libraries and termed Sandworm herpesvirus (1971 bp) and Peanut worm herpesvirus (13,115 bp). Peanut worm herpesvirus had highly abundant reads in the BHNXC library (0.01% of non-rRNA reads) sourced from peanut worms. Sandworm herpesvirus, from sandworms, was relatively abundant (0.0002%) in the BHSC library (Table 1). Both of these herpes-like virus sequences contained regions of high similarity to DNA-packaging terminase protein from other members of Herpesviridae. When aligned against the amino acid sequence of DNA-packaging terminase protein 1 from members of the Herpesviridae (*n* = 37) these sequences were highly divergent, with only ~30% identity to other invertebrate herpesviruses. In a phylogenetic tree inferred from this alignment, both sequences fell basal to gamma-, alpha- and beta-herpesviruses, but were distinct from mollusc-infecting malacoherpesviruses (Figure 4A).

#### 3.3.2. *Polyomaviridae*

The Spider library (Table 2) yielded a polyoma-like virus sequence (2281 bp). The sequence obtained, termed Spider polyomavirus, possessed 35.2% amino acid identity to Trichodysplasia spinulosa-associated polyomavirus large T-antigen protein (Table 1). A large T antigen-like ORF from the sequence was used in an alignment with polyomavirus large T-antigen proteins (*n* = 84), and a phylogenetic tree of this alignment reveals that Spider polyomavirus is highly divergent, with its closest relative being the single other invertebrate infecting polyomavirus, Baja California bark scorpion polyomavirus 1 (Figure 4B).

#### 3.3.3. *Nimaviridae*

The Shrimp library (Table 2) yielded a nima-like sequence (837 bp) that was relatively abundant (0.0001%). The nima-like sequence exhibited 88.5% amino acid identity to the anti-apoptosis protein in WSSV (Table 1). The nima-like sequence has a highly-conserved anti-apoptosis protein (wsv390) that is 94%–100% similar to six WSSV anti-apoptosis protein sequences (Supplementary Figure S2b), with all the diversity located in a low-complexity region the 14 bp of the N termini of the protein (Supplementary Figure S2c). The results from a nucleotide BLAST of this novel nima-like sequence shows that the 837 bp fragment is very similar (99%) to many other WSSV sequences. Because of high degree of sequence similarity, clearly evident from the phylogenetic analysis (Supplementary Figure S2a), we named this WSSV variant Zhejiang, although it may represent an already characterized WSSV.

#### 3.3.4. *Nudiviridae*

The BHXun library (sampled from sesarmid crabs, Table 2) yielded two highly divergent nudi-like sequences, Charybdis crab nudivirus 1 and Charybdis crab nudivirus 2 (1054 bp and 1168 bp), that were related (~50% amino acid identify) to the DNA polymerase proteins from other crustacean-infecting nudiviruses (Table 1). Both sequences were relatively abundant within the BHXun library: 0.0005% and 0.0007% for Charybdis crab nudivirus 1 and Charybdis crab nudivirus 2, respectively. As the two sequences were homologous to different regions of the amino acid sequence of the highly conserved DNA polymerase (Supplementary Figure S1), we aligned them separately with the three other crusteacean-infecting nudiviruses available on GenBank and from this estimated two phylogenetic trees (Supplementary Figure S1). Interestingly, the two new nudiviruses fell in similar positions, close to Homarus gammarus nudivirus, that infects European lobsters, and separate from Helicoverpa zea nudivirus and Heliothis zea nudivirus.

## 4. Discussion

Using a meta-transcriptomics approach that does not selectively target viral particles, we identified a number of novel of single-stranded and double-stranded DNA viruses that broadens our perspective on the diversity and evolution of invertebrate DNA viruses. ssDNA viruses are the smallest encapsulated infectious agents, present in all domains of life, and likely of great antiquity [29,30]. In comparison to many dsDNA viruses that often encode polymerases and complex arrays of proteins, ssDNA viruses have smaller genomes (often less than 6 kb) and rely on host proteins for their replication [31]. Although recent metagenomic studies have resulted in the discovery of many novel ssDNA viruses, issues such as reagent contamination and low protein/nucleotide similarity has complicated analysis and classification [18,19,32]. Currently, the ICTV recognises 11 families of ssDNA viruses, with several new families discovered recently including the *Genomoviridae, Redondoviridae*, and *Smacoviridae* [13,15,33]. Known invertebrate ssDNA viruses include members of the *Parvoviridae, Circoviridae*, and *Bidnaviridae*, and recent metagenomic studies have recently identified ssDNA in a variety of invertebrates, including termites [34], pine beetles [35] honey bees [36], dragonflies [6], and a range of marine and terrestrial invertebrates [9,37]. Similarly, CRESS DNA viruses, that contain many ssDNA virus families (including the *Genomoviridae, Smacoviridae, Bacilladnaviridae*, and *Circoviridae*), have also been sampled from invertebrates [33], as well as from such diverse environments as oceans and estuaries [38,39] and even poxvirus lesions in tanagers [31]. We add to this diversity by identifying novel species and variants of previously identified species of ssDNA viruses from the *Circoviridae*, *Genomoviridae*, and *Parvoviridae*.

Members of *Circoviridae* are ~1.8–2.1 kb in length, have antisense genomes, and possess non-enveloped virons [40,41,42]. Circoviruses have mainly been identified in mammalian or avian hosts, although this has expanded with the advent of metagenomic studies [43,44,45,46,47,48,49], and circular ssDNA genomes have also been identified in animal and environmental samples [50,51]. Rosario and colleagues made the first discovery in invertebrates with the identification of a novel cyclovirus in dragonflies [6,52], with later studies demonstrating the presence of these viruses in other invertebrates [53]. Cycloviruses share many characteristics with circoviruses, although they occupy a distinct phylogenetic position and can infect both vertebrates and invertebrates [14]. Herein, we identified three novel members of the *Circoviridae*, with two cycloviruses; Flea cyclovirus and Arthropod cyclovirus, and a single novel circovirus, Sandworm circovirus, that falls within the circovirus clade. 

An important complicating factor in the analysis of the *Circoviridae*, and of ssDNA viruses in general, is that they appear to be ubiquitous contaminants of commonly used laboratories reagent [18,19]. It is therefore crucial to exercise caution when assigning novel circoviruses, ensuring that they represent bona fide viruses and not those present in reagents. To help identify potential contaminants in our phylogenetic analysis, we included a circo-like virus sequence from sterile H_2_O obtained from a previous experimental blank (Table 1). Notably, this sequence, termed “Blank” falls within a diverse range of circoviruses that are distinct from the cycloviruses and the vertebrate-infecting circoviruses. These include many viruses derived from marine or faeces-associated environments, indicating that these are of uncertain provenance and may be derived from an environmental source during spin-column manufacturing [54]. Similarly, circovirus-like sequences previously suggested to be reagent contaminants [18] also fall within these divergent viral clades. Although there is a diverse range of laboratory contaminant-associated circoviruses, including our Blank sequence, that necessitates caution, the two novel sequences that fall within the cyclovirus clade, Flea cyclovirus and Arthropod cyclovirus, are likely genuine novel cycloviruses. As Flea cyclovirus was found in Myriapoda and Arthropod hosts, the most likely host is millipedes, although this awaits confirmation. Arthropod cyclovirus was found in a library comprising various insect hosts that complicates host classification. Sandworm circovirus was identified in a sandworm library and is phylogenetically divergent. However, on these data it is difficult to determine whether the novel Sandworm circovirus is associated with sandworms or laboratory reagents, and it falls in the same clade as a Dromedary stool associated circovirus that likely represents a reagent contaminant. Circo-like sequences were identified in many libraries (Figure 1B, abundance shown in purple) and make up a large proportion of the DNA virus-like sequences in their respective libraries. Importantly, that they were not found in all libraries suggests that there was not generalised contamination throughout our reagents. This is consistent with the high sample biomass used for all extractions and library preparations that will limit signal from less abundance reagents contaminants. We are aware that this issue requires further investigation, and we will explore reagent contamination more thoroughly in the future (Porter et al., in preparation).

The *Genomoviridae* are a relatively newly described family of eukaryotic-infecting ssDNA viruses, first described in 2016 [13]. One of the nine genera in the family, *Gemykibivirus*, contains the only ssDNA virus known able to infect fungi, Sclerotinia sclerotiorum hypovirulence-associated DNA virus 1 (SsHADV-1) [55]. SsHADV-1 is a non-enveloped, isometric, 20–22 nm virion, with a ~2 kb genome that encodes 2 genes (replication-associated protein and capsid protein) [13]. Other members of *Genomoviridae* are associated with faeces or sewerage [13], pine beetles [34], termites [33], and even bird nests [56], again complicating the identification of the true host. It is possible that some members of *Genomoviridae* are present in reagents, as is the case with the *Circoviridae.* Herein, we identified three new members of Gemykibivirus, a genus within the *Genomoviridae.* This not only expands the known diversity of a relatively new family of viruses, but demonstrates the potential ability of viruses in *Genomoviridae* to infect Myriapoda that contain millipedes, centipedes and other terrestrial species. Further identification of novel members of *Genomoviridae* will undoubtedly help reveal the true hosts. 

The *Parvoviridae* infect a wide range of invertebrates and vertebrates, including humans. Members of the *Parvoviridae* family have compact, linear genomes of ~5 kb that only encode two genes [57] and require hosts for most cellular processes, including to initiate S phase to synthesize the first viral strands [58,59,60]. To overcome this, some parvoviruses co-infect with helper viruses [58,61], while others are adapted to infect cell types that undergo S phase in their cell cycle. There are two subfamilies of *Parvoviridae*, *Densovirinae* and *Parvovirinae*, associated with invertebrates and vertebrates, respectively. Members of the *Densovirinae* are known to infect insects, crustaceans and echinoderms, and currently comprise five genera: *Ambidensovirus, Brevidensovirus, Hepandensovirus, Iteradensovirus*, and *Penstyldensovirus* [62]. We anticipated that if any invertebrate parvoviruses identified here would be from the invertebrate-associated *Densovirinae*. In confirmation, we identified seven densoviruses, including new potential hosts such as sesarmid crabs, spiders, and fleas, although some are very closely related to previously identified densoviruses. In contrast, one of the novel densoviruses discovered, Tetragnatha maxillosa densovirus, is highly divergent within the phylogenetic tree (Figure 2B). This suggests that there may be a greater diversity of densovirus hosts than what has been currently sampled, and we suggest that further investigation into insect hosts will likely identify many more novel densoviruses. Several densovirus sequences that are likely reagent contaminants [18] were included in this analysis but were not closely related to the novel densoviruses identified here, such that these novel viruses may indeed infect the invertebrates from which they were sampled.

Notably, these meta-transcriptomic data also contain a number of dsDNA viruses. Currently, the International Committee on Taxonomy of Viruses (ICTV) classifies dsDNA viruses into 25 families (ICTV; URL: http://www.ictvonline.org) that range in size from <10 kb to >150 kb. Three virus groups are known to infect invertebrates: (i) nucleo-cytoplasmic large DNA viruses, (ii) mollusc-infecting herpesviruses, and (iii) large rod-shaped nuclear replicating DNA viruses, that includes white spot syndrome virus. WSSV, a member of the *Nimaviridae*, poses a serious economic threat to the fishing industry and aquaculture [63], impacting both cultured shrimp and crustaceans in both freshwater and marine environments [64,65]. We identified five novel dsDNA virus sequences and a sequence of WSSV in the Shrimp library (Supplementary Figure S3), which may constitute a new variant of this virus (variant Zhejiang). 

Two novel nudi-like sequences were identified in the BHXun library (Supplementary Figure S2). As these two sequences aligned to different regions of the highly-conserved DNA polymerase protein, we inferred two separate phylogenetic trees, although in both cases they fall close to Homarus gammarus nudivirus that infects European lobsters, and other members of *Nudiviridae* are known to infect crustaceans. As the BHXun library was sampled from sesarmid crabs, we suggest that these two novel nimavirus sequences, termed Charybdis crab nudivirus 1 and Charybdis crab nudivirus 2, are new members of *Nudiviridae* that also infect crustaceans.

Two novel herpes-like sequences were identified in the BHSC and BHNXC libraries. Most *Herpesvirales* identified are pathogenic to vertebrates, with the exception of members of the *Malacoherpesviridae* that infect molluscs. We used sequences of the highly conserved DNA packaging ATPase subunit of terminase protein [66] to determine the phylogenetic position of the two novel herpes-like sequences identified here: accordingly, Sandworm herpesvirus and Peanut worm herpesvirus fall between the family *Malacoherpesviridae* and the main three subfamilies of *Herpesviridae* (alpha, beta, gamma), although they are also highly divergent from each other. We therefore suggest that other invertebrate-infecting members of *Herpesvirales* will be identified with increased sampling, broadening our understanding of his important group of viruses.

At present, only one known invertebrate-infecting polyomavirus has been identified, Baja California bark scorpion polyomavirus 1. Other known members of the *Polyomaviridae* infect mammals and birds, and appear to have experienced a long period of co-evolution with their hosts [67]. We identified a novel and highly divergent polyoma-like large T antigen virus protein sequence in *Trichodysplasia spinulosa* spider samples and termed Spider polyomavirus (Figure 4B). While the novel Spider polyomavirus is most closely related to Baja California bark scorpion polyomavirus 1, these two viruses are still highly divergent from each other. This suggests that the *Polyomaviridae* may contain many more members capable of infecting invertebrates, perhaps as diverse as vertebrate-infecting polyomaviruses.

As invertebrates have complex ecological interactions with both plants and vertebrates, the study of invertebrate viruses provides a valuable insight on the ecological and evolutionary factors that shape viral diversity and the host-pathogen interactions. We revealed unknown diversity of DNA viruses in invertebrates, particularly in the *Circoviridae, Genomoviridae, Polyomaviridae*, and *Herpesviridae*, for which few, invertebrate hosts described previously. From these results, it is easy to hypothesize that there are many other, as yet undescribed, DNA viruses present in invertebrates. To fully understand this group of diverse viruses the thorough sequencing of a wide variety of invertebrate species is required, although this is complicated by a lack of conserved regions, low viral load, large genomes and the presence of closely related endogenous genes. A limitation of meta-transcriptomics in comparison to approaches such as such as cell culture, serology and PCR, is that it cannot exclude the possibility of co-infecting microorganisms within the host, confounding the identification of the true host of any viruses. However, unlike traditional approaches, meta-transcriptomics has the potential to determine the entire virome infecting a host, including highly divergent, low-abundance, and potentially novel viruses, and to do so in an unbiased manner, providing a useful tool for both RNA and DNA virus discovery.

## Figures and Tables

**Table 1:** Summary of the candidate DNA viruses identified in this study.

	***Family***	***Virus Name***	***Library***	***Length***	NR * Protein Hit	NR * Protein Hit Name and Species	NR * Hit Percentage (%) Amino Acid Identity	NR * Hit Length	NR * e-Value	***Taxonomy as Identified by Taxonomist (Family, Order, Genus)***	***Conserved Domain ID***	***Conserved Domain Name***	***Conserved Domain e-Value***	Abundance in Library (%) **
***ssDNA***	*Parvoviridae*	Tetragnatha maxillosa densovirus	SSZZ	1087	YP_164339.1	Nonstructural protein 1 [Dendrolimus punctatus densovirus]	28.7	157	4.40 × 10^−11^	*Parvoviridae, Densovirinae, Iteradensovirus*	329234	Parvo_NS1	2.50 × 10^−15^	0.002
		Perisesarma bidens hepandensovirus	SCJXSX	940	YP_003799994.1	Non-structural protein 1 [Fenneropenaeus chinensis hepandensovirus]	59	156	5.10 × 10^−48^	*Parvoviridae, Densovirinae, Hepandensovirus*				0.0002
		Periplaneta fuliginosa densovirus variant Hubei	WHZM	1658	NP_051017.1	Structural protein [Periplaneta fuliginosa densovirus]	100	353	1.50 × 10^−204^	*Parvoviridae, Densovirinae, Ambidensovirus*	280494	Denso_VP4	0	0.27
		Arthropod densovirus	WHCC	970	AJD76764.1	NS1-like protein 2 [Penaeus stylirostris densovirus]	41.8	170	2.30 × 10^−27^	*Parvoviridae, Densovirinae*	329234	Parvo_NS1	8.91 × 10^−14^	0.0001
		Decapod penstyldensovirus 1 variant Zhejiang	Shrimp	3137	AGO44085.1	Nonstructural protein 1 [Decapod penstyldensovirus 1]	94.6	723	0	*Parvoviridae, Densovirinae, Penstyldensovirus*	329234	Parvo_NS1	9.36 × 10^−09^	0.02
		Mydriapoda densovirus 1	WGML	739	AAT35223.1	Nonstructural protein 1 [Haemagogus equinus densovirus]	46.6	249	7.30 × 10^−58^	*Parvoviridae, Densovirinae, Brevidensovirus*				0.00006
		Myridapoda densovirus 2	WGML	2460	AAT35223.1	Nonstructural protein 1 [Haemagogus equinus densovirus]	43.5	481	2.50 × 10^−102^	*Parvoviridae, Densovirinae, Brevidensovirus*	329234	Parvo_NS1	2.55 × 10^−05^	0.001
		Flea cyclovirus	WHZM	1602	AEL87792.1	Putative replication-associated protein [Bat circovirus ZS/China/2011]	97.9	286	8.40 × 10^−168^	*Circoviridae, Cyclovirus*	280553	Viral_Rep	7.27 × 10^−16^	0.87
***ssDNA***	*Circoviridae*	Sandworm circovirus	BHSC	1063	YP_009116906.1	Replication-associated protein [Sewage-associated circular DNA virus-21]	41.5	236	5.80 × 10^−40^	NA	280553	Viral_Rep	1.02 × 10^−09^	0.0004
		Arthropod cyclovirus	ZCM	1911	AIZ46815.1	Replication associated protein [Swine cyclovirus]	77.4	221	3.80 × 10^−98^	*Circoviridae, Cyclovirus*	280553	Viral_Rep	0.00136865	0.02
	***Family***	***Virus name***	***Library***	***Length***	NR * protein hit	NR * protein hit name and species	NR * hit percentage	NR * hit length	NR * e-value	***Taxonomy as identified by Taxonomist (Family, Order, Genus)***	***Conserved domain ID***	***Conserved domain name***	***Conserved domain e-value***	Abundance in library (%) **
	*Genomoviridae*	Myriapoda gemycircularvirus 1	WGML	4487	YP_009109722.1	Capsid protein [Faeces-associated gemycircularvirus 8]	66.3	264	5.00 × 10^−101^	*Genomoviridae, Gemykibivirus*		Gemini_AL1	6.57 × 10^−10^	0.003
		Myriapoda gemycircularvirus 2	WGML	4571	YP_009259554.1	Replication initiation associated protein [Gemycircularvirus HV-GcV2]	49.3	343	1.50 × 10^−84^	*Genomoviridae, Gemykibivirus*		Gemini_AL1	3.30 × 10^−18^	0.0005
		Myriapoda gemycircularvirus 3	WGML	4648	YP_009259554.1	Replication initiation associated protein [Gemycircularvirus HV-GcV2]	46.9	356	1.30 × 10^−83^	*Genomoviridae, Gemykibivirus*		Gemini_AL1	2.90 × 10^−13^	0.005
***dsDNA***	*Polyomaviridae*	Spider polyomavirus	Spider	2281	AID54935.1	Large T antigen [Trichodysplasia spinulosa-associated polyomavirus]	35.2	244	1.20 × 10^−26^	*Polyomaviridae, Alphapolyomavirus*	333065	PHA03102	1.92 × 10^−40^	N/A
	*Nimaviridae*	White spot syndrome virus variant Zhejiang	Shrimp	837	YP_009220600.1	Anti-apoptosis protein [White spot syndrome virus]	88.5	139	2.90 × 10^−63^	*Nimaviridae, Whispovirus*	329062	TK	3.83 × 10^−17^	0.0001
	*Nudiviridae*	Charybdis crab nudivirus 1	BHXun	1054	YP_009051843.1	DNA polymerase [Penaeus monodon nudivirus]	48.5	342	4.70 × 10^−90^	*Nudiviridae*	324564	POLBc	7.10 × 10^−10^	0.000553059
	*Nudiviridae*	Charybdis crab nudivirus 2	BHXun	1168	YP_009051843.1	DNA polymerase [Penaeus monodon nudivirus]	52.6	371	6.90 × 10^−103^	*Nudiviridae*	332123	POLBc	3.63 × 10^−12^	0.000721381
	*Herpesviridae*	Sandworm herpesvirus	BHSC	1971	AMW36220.1	Putative DNA packaging terminase [Abalone herpesvirus Taiwan/2005]	33.9	254	9.50 × 10^−28^	*Herpesvirales*	333071	AddB	0.011	0.000222584
		Peanut worm herpesvirus	BHNXC	13115	YP_009229862.1	DNA packaging terminase subunit 1 [Myotis gammaherpesvirus 8]	25.6	429	9.40 × 10^−23^	*Herpesviridae, Gammaherpesvirinae*	330553	SMC_N	0.000744176	0.018654163

* NR = non-redundant protein NCBI database; ** From all reads in the library excluding rRNA.

**Table 2:** Host classification and geographic location of the invertebrate samples utilised here.

Library	Name	Host Classification	Species of Host (No. Individuals)	Sample Type	Habitat	Data Generated	Library Accession
Hubei Myriapoda mix	WGML	Arthropoda: Myriapoda	*Diplopoda sp. (7), Otostigmus scaber (4), Scolopocryptops sp.* *(3), Otostigmus scaber (1), Myriapoda sp. (1).*	Whole individual	Hubei (Terrestrial)	7,337,861,800	SRX1029951
Hubei flea and ant mix	WHZM	Arthropoda: Insecta	*Ctenocephalides felis (10-15), Tetramorium tsushimae (N/A)*	Whole individual	Hubei (Terrestrial)	1,680,999,000	SRR3400840
Spiders	Spider	Arthropoda: Chelicerata	*Neoscona nautica (14), Parasteatoda tepidariorum (3), Plexippus setipes (3), Pirata sp. (1), Araneae sp. (8)*	Whole individual	Hubei (Terrestrial)	11,361,912,300	SRX833697
Insect Mix 4 (described previously)	WHCC	Arthropoda	*Pseudothemis zonata (2), Nepidae sp. (2 species, 3), Camponotus japonicas (~5), Diplonychus sp. (2), Asellus sp. (N/A)*	Whole individual	Hubei (Terrestrial)	11,973,368,200	SRX833688
Shrimps	Shrimp	Arthropoda: Crustacea	*Exopalaemon carinicauda (12), Metapenaeus sp. (6), Solenocera crassicornis (6), Penaeus monodon (12), Litopenaeus vannamei (12)*	Representative tissues from gill, various glands, muscle, and gut	Zhejiang (Costal/Marine)	5,365,359,900	SRX833698
*Tetragnatha maxillosa* mix Hubei (subset of arthropodmix)	SSZZ	Arthropoda: Chelicerata	*Tetragnatha maxillosa (2)*	Whole individual	Hubei (Land/Freshwater)	2,933,255,400	SRR3401144
Sesarmid crab mix Beihai	SCJXSX	Arthropoda: Crustacea	*Perisesarma bidens (8)*	Representative tissues from gill, various glands, muscle, and stomach (cleared)	Guangxi (Costal/Marine)	6,176,296,400	SRR3401386
Turritella sea snails mix Beihai	BZL	Mollusca	*Turritella sp.(12)*	Visceral mass with gut content removed	Guangxi (Costal/Marine)	5,294,621,400	SRX1712841
Orthoptera mix Hubei	ZCM	Arthropoda: Insecta	*Orthoptera sp. (3), Conocephalus sp. (4), Gryllidae sp. (3)*	Whole individual	Hubei (Terrestrial)	7,042,417,800	SRX1029952
Sandworms mix Beihai	BHSC	Annelida	*Marphysa sp. (12), Nereis aibuhitensis (12)*	Whole individual	Guangxi (Costal/Marine)	6,114,388,200	SRX1712659
Millipedes	WHWG	Arthropoda: Myriapoda	*Polydesmidae sp. (2 species, 12 individuals)*	Whole individual	Hubei, Beijing (Terrestrial)	7,176,702,400	SRX833700
Charybdis crab mix Beihai	BHXun	Arthropoda: Crustacea	*Charybdis sp. (12)*	Representative tissues from gill, various glands, muscle, and stomach (cleaned)	Guangxi (Costal/Marine)	6,439,342,400	SRR3401303
Arthopod mix Hubei	Arthropodmix	Arthropoda	*Cicadellidae sp. (10), Tetragnatha maxillosa (1), Heteroptera sp. (10), Scutigeridae sp. (2), Ctenocephalides felis (10-15), Tetramorium tsushimae (N\A)*	Whole individual	Hubei (Terrestrial)	7,768,505,600	SRX1029954
Peanut worms mix Beihai	BHNXC	Sipuncula	*Phascolosoma esculenta (12), Sipunculus nudus (12)*	Whole individual	Guangxi (Costal/Marine)	7,041,903,800	SRR3401570

## Supplementary Materials

The following are available online at https://www.mdpi.com/1999-4915/11/12/1092/s1, Figure S1: Visualising the relationships of three amino acid sequences of the highly conserved protein DNA polymerase from the *Nudiviridae*, including the two novel nudiviruses found in this study. Figure S2: Visualising the relationships of five known amino acid sequences of highly conserved anti-apoptosis protein from the *Nimaviridae*, including the potentially novel Zhejiang variant identified here. Figure S3: Visualisation of the amino acid and nucleotide level similarity of novel Decapod penstyldensovirus 1 variant Zhejiang with other sequences of the same species. Table S1: Additional libraries included in this study. Table S2: The amino acid substitution used in the phylogenetic analysis.
