# Supplementary material for: Diversity and Evolution of Novel Invertebrate DNA Viruses Revealed by Meta-Transcriptomics
Source: Viruses. 2019 Nov 25;11(12):1092. doi: 10.3390/v11121092 (PMC6950620; doi:10.3390/v11121092)
Supplement: Supplementary file 1 [file viruses-11-01092-s001.pdf]

Supplementary Materials

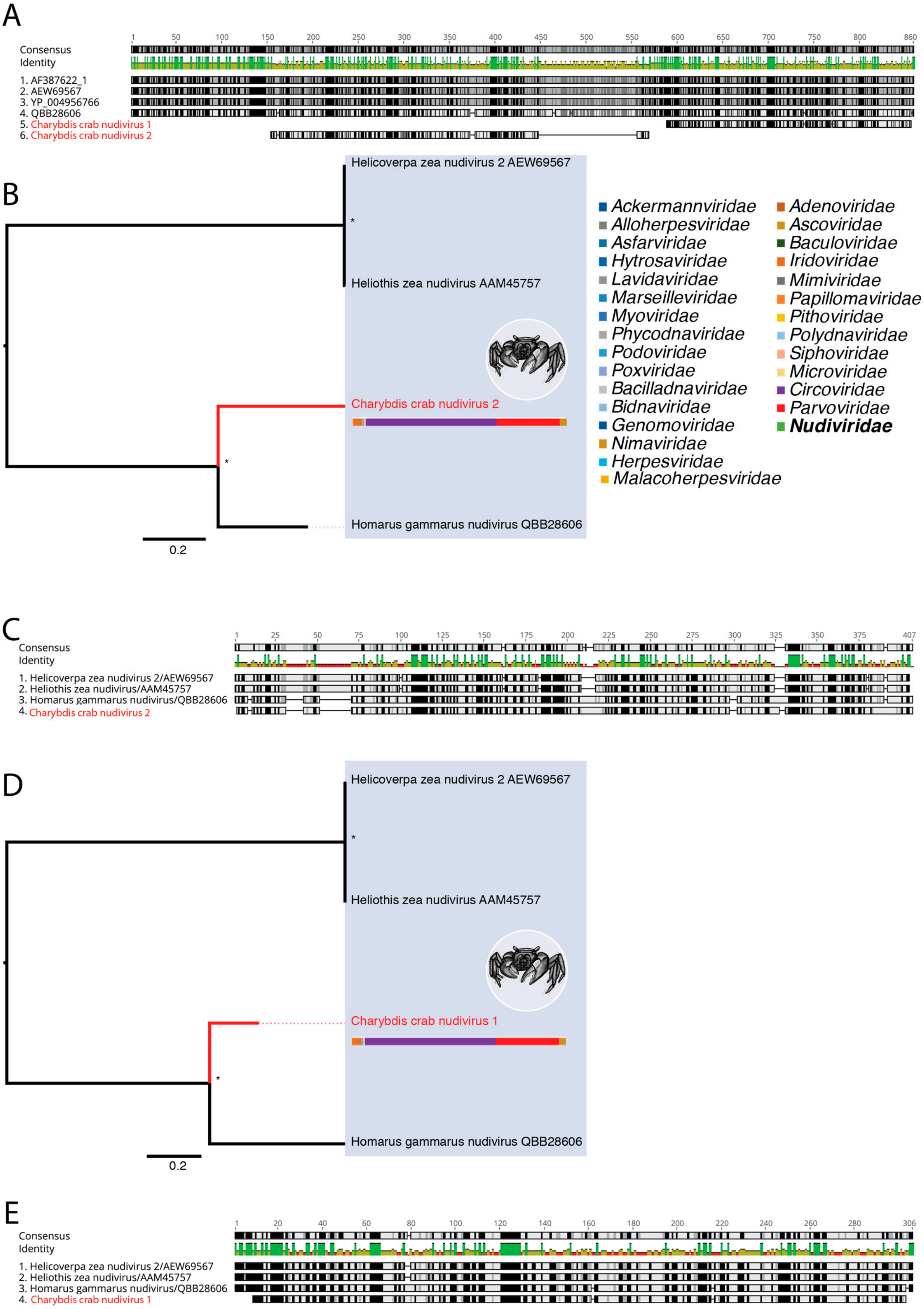

Figure S1. Visualising the relationships of three amino acid sequences of the highly conserved DNA polymerase protein from the *Nudiviridae,* including the two novel nudiviruses identified here. **(A)** An alignment of the two novel nudivirus sequences with three known members of the *Nudiviridae*. As the two novel sequences aligned to different regions of DNA polymerase, separate phylogenetic trees were estimated for each sequence. **(B)** The phylogenetic relationships among the nudiviruses and Scylla nudivirus (highlighted in red) from the BHXun library (from sesarmid crabs). **(C)** The alignment of Scylla nudivirus and other members of *Nudiviridae* used in the phylogenetic analysis. **(D)** The phylogenetic relationship among nudiviruses and Charybdis nudivirus (highlighted in red), from the BHXun library. **(E)** Alignment of Charybdis nudivirus and other members of *Nudiviridae* used in the phylogenetic analysis. The abundance of nudi-like contigs (in green) in the BHXun library is shown next to each novel species. Bootstrap values greater than 85% are marked by an asterisk. For clarity, the trees were mid-point rooted, with all horizontal branch lengths scaled according to the number of amino acid substitutions per site.

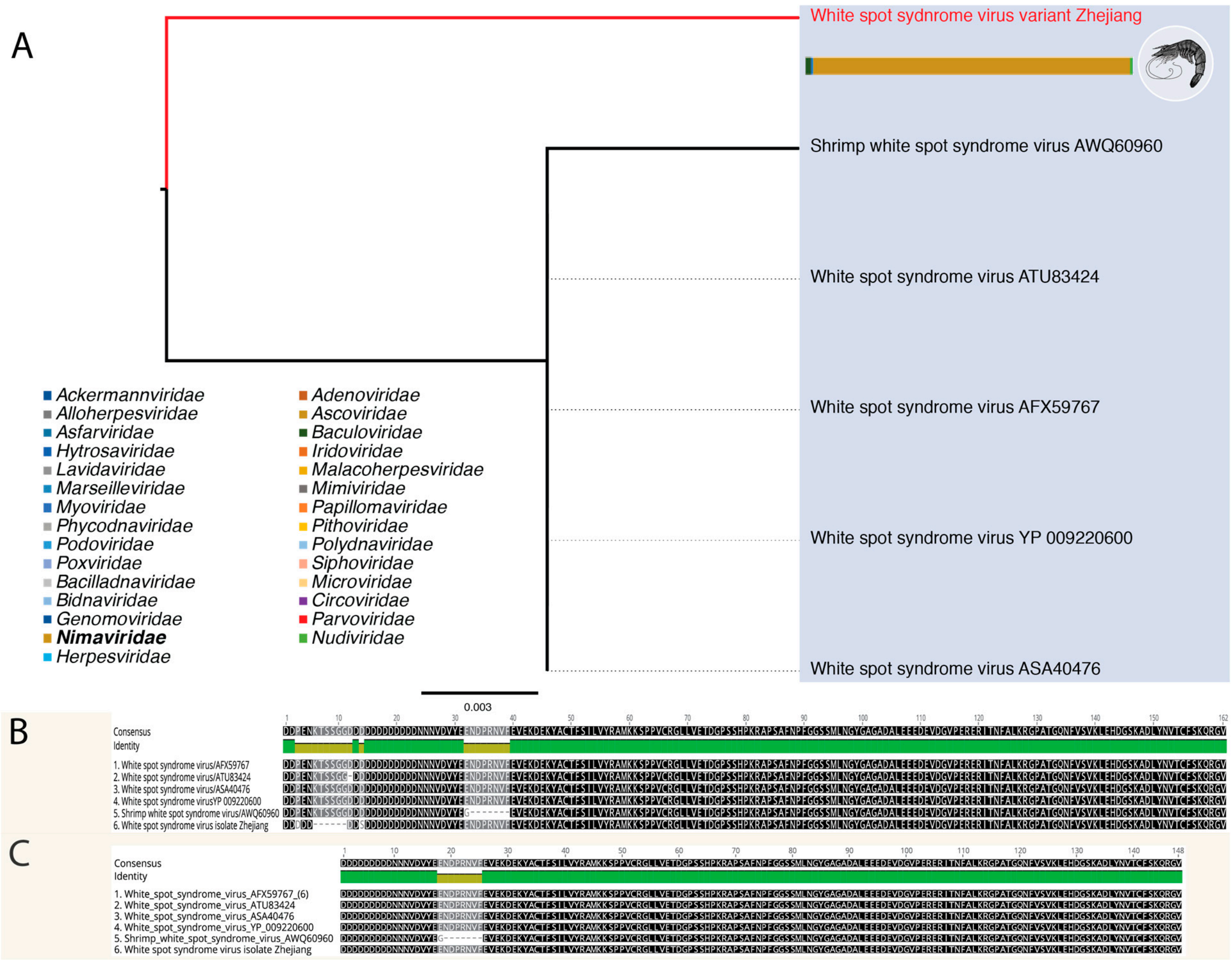

Figure S2. Visualising the relationships of five known amino acid sequences of the highly conserved anti-apoptosis protein from the *Nimaviridae*, including the potentially novel WSSV variant (Zhejiang) identified in this study. **(A)** Amino acid sequences of the conserved anti-apoptosis protein in the family *Nimaviridae* (n = 5) used in an alignment with the ORF hypothesised to be WSV390 in the novel WSSV variant Zhejiang. **(B)** Phylogenetic relationships of WSSV sequences, including WSSV variant Zhejiang (in red). The abundance of nima-like contigs (yellow) in the Shrimp library is shown beside WSSV variant Zhejiang. Only bootstrap values greater than 85% are shown. All horizontal branch lengths are scaled according to the number of amino acid substitutions per site and the tree was mid-point rooted for clarity.

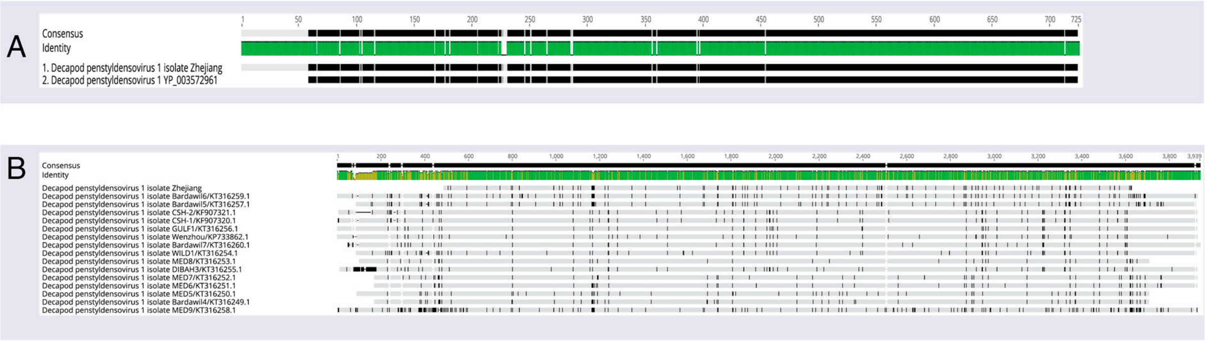

Figure S3. Visualisation of the amino acid and nucleotide level similarity of novel Decapod penstyldensovirus 1 variant Zhejiang with other sequences of the same species. (**A**) Amino acid alignment of the NS1 protein of novel Decapod penstyldensovirus 1 variant Zhejiang with Decapod penstyldensovirus 1. (**B**) Nucleotide alignment of the whole genome of Decapod penstyldensovirus 1 variant Zhejiang and other members of the same species (n = 15).

Table S1. Additional libraries included in this study.

Name	*Library*	*Host*	*Habitat*	*Library accession*
YC	Plasmodium mix	*Plasmodium berghei*	Hubei	
WZGZ	Maxillopoda mix	Maxillopoda	Wenzhou	SRR10012027
CPQYII	Earthworm mix Changping II	Oligochaeta	Beijing	
BZLII	Turritella sea snails mix Beihai II	Turritella	Beihai	
BHRJ	Beihai arthropod mix	Arthropoda	Beihai	

Table S2. The amino acid substitution models employed in the phylogenetic analysis performed here.

**Virus family**	**Model**	Γ	**I**	**F**
Circoviridae	RtRev	x		x
Parvoviridae	RtRev	x		x
Gemycircularviridae	RtRev	x	x	x
Nimaviridae	RtRev			x
Polyviridae	LG	x	x	x
Nudiviridae	RtRev	x		x
Herpesviridae	LG	x	x	
